# Whole genome sequencing analysis of high confidence variants of B-cell lymphoma in *Canis familiaris*

**DOI:** 10.1371/journal.pone.0238183

**Published:** 2020-08-28

**Authors:** Alana Sparks, J. Paul Woods, Dorothee Bienzle, Geoffrey A. Wood, Brenda Lynn Coomber

**Affiliations:** 1 Department of Biomedical Sciences, Ontario Veterinary College, University of Guelph, Guelph, ON, Canada; 2 Department of Clinical Studies, Ontario Veterinary College, University of Guelph, Guelph, ON, Canada; 3 Department of Pathobiology, Ontario Veterinary College, University of Guelph, Guelph, ON, Canada; University of Bologna, ITALY

## Abstract

Lymphoma (lymphosarcoma) is the second most frequent cancer in dogs and is clinically comparable to human non-Hodgkin lymphoma. Factors affecting canine lymphoma progression are unknown and complex, but there is evidence that genetic mutations play an important role. We employed Next Gen DNA sequencing of six dogs with multicentric B-cell lymphoma undergoing CHOP chemotherapy to identify genetic variations potentially impacting response. Paired samples from non-neoplastic tissue (blood mononuclear cells) and lymphoma were collected at the time of diagnosis. Cases with progression free survival above the median of 231 days were grouped as ‘good’ responders and cases below the median were categorized as ‘poor’ responders. The average number of variants found was 17,138 per case. The variants were filtered to examine those with predicted moderate or high impacts. Many of the genes with variants had human orthologs with links to cancer, but the majority of variants were not previously reported in canine or human lymphoma. Seven genes had variants found in the cancers of at least two ‘poor’ responders but in no 'good’ responders: *ATRNL1*, *BAIAP2L2*, *ZNF384*, *ST6GALNAC5*, *ENSCAFG00000030179* (human ortholog: riboflavin kinase RFK), *ENSCAFG00000029320*, and *ENSCAFG00000007370* (human ortholog: immunoglobin IGKV4-1). Two genes had variants found in the cancers of at least two 'good’ responders but in no 'poor’ responders: *COX18* and *ENSCAFG00000030512*. *ENSCAFG00000030512* has no reported orthologue in any other species. The role of these mutations in the progression of canine lymphoma requires further functional analyses and larger scale study.

## Introduction

Lymphoma (lymphosarcoma) originates from poorly controlled lymphocyte clonal expansion, and is the second most frequent canine cancer, accounting for 90% of all hematopoietic cancers in dogs [1–3]. Canine lymphoma (CL) usually involves primary and secondary lymphoid tissues, including lymph nodes, spleen, bone marrow and thymus, and may spread to the skin, intestinal tract, liver, eye, central nervous system and bone [4]. CL is generally regarded as being highly similar to human non-Hodgkin lymphoma (NHL) [5]. Both cancers occur spontaneously, have similar clinical presentation and pathophysiology, along with a closely parallel natural progression and response to chemotherapy [3].

Dogs have a compressed lifespan compared to humans and a faster aging process, making clinical studies in dogs shorter and less expensive than in humans [6]. Dogs are a prominent pet in many societies, share similar environments to humans, have well-recorded health data, and have owners who are willing to seek and fund treatment [3]. Cancer treatment modalities are very similar in humans and dogs, and include surgery, radiation, corticosteroids, cytotoxic chemotherapy, and molecularly targeted therapy [1]. Due to the similarities between CL and NHL, any findings in CL can be helpful in the study of NHL.

Diagnosis of CL is done via histopathology and/or cytology, often with the aid of immunohistochemistry, and immunophenotyping is usually done by flow cytometry [7, 8]. The current standard of care is a doxorubicin-based multidrug chemotherapy protocol known as CHOP; cyclophosphamide, hydroxydaunorubicin, vincristine (Oncovin^®^), and prednisone [3, 9]. Most dogs with lymphoma are not cured but go into remission of varying duration. Many dogs have their cancer managed with chemotherapy for a median period of 7–10 months and live for a median period of 10–14 months after diagnosis [3]. While there is a high initial response rate with CHOP, the majority of dogs will relapse and succumb to their disease within 2 years, often due to development of drug resistance [9].

The causes of CL are unknown and complex. CL can affect any breed at any age, although it is more common in larger breeds and in older dogs [3]. Some breeds that appear to be disproportionately affected include: Boxers, Scottish terriers, Airedale terriers, Basset hounds, German shepherds, Bulldogs, and Bernese Mountain dogs [7]. Some breeds also appear to be predisposed to certain immunophenotypes of CL: *e*.*g*. Boxers and T-cell lymphoma, and German shepherds and B-cell lymphoma [7]. When comparing exomes across various breeds, there was some overlap in significantly mutated genes in B-cell lymphoma but no overlap with cases of T-cell lymphoma [10]. This finding suggests that B-cell lymphomas from different breeds have common genetic causes, while T-cell lymphomas have mutations that tend to be breed-specific [10].

Whole genome studies have been applied to many diseases in recent years. Efforts to identify genetic components of lymphoma have focused on human, mouse, and dog models. Studies in humans have identified *TP53* as a possible site involved in the initiation of NHL and many mutations in *TP53* have been found in CL as well [10, 11]. Hundreds of other mutations have also been associated with the initiation and progression of CL through whole genome analysis [5, 10, 12]. Pathways affected in NHL include chromatin modification, NF-kB, phosphoinositide 3-kinase, B-cell lineage, and Wnt signaling. The genetic changes associated with CL include chromosomal gains, losses, deletions, amplifications and duplications [3, 5, 10, 12].

Here we examine the genomes of six dogs, with paired samples from a non-neoplastic tissue (blood mononuclear cells) and a sample of cancerous tissue (needle aspirate of enlarged peripheral lymph node) to identify significant genetic variations in neoplastic tissue relative to non-neoplastic tissue. We identified multiple genetic changes, both in the tumour samples and in the buffy coat samples relative to the canine reference genome. Several of these gene mutations were previously described, but many are novel and their potential role in CL progression warrants further investigation.

## Materials and methods

### Study subjects

The subjects were recruited for a study testing biomarkers as predictors for chemotherapy efficacy in CL patients. This study was conducted with approval of the University of Guelph Animal Care Committee (AUP #1442–007), under the guidelines of the Canadian Council on Animal Care (CCAC). There was no cost to the owner to participate in the study and all owners provided informed consent for their dogs to participate. The eligibility criteria for the study included: multicentric lymphoma with enlarged lymph nodes, intent to treat with multiagent CHOP chemotherapy, and no previous or current cancer diagnosis other than lymphoma. Of the six subjects enrolled in this study, one was a German Shepherd, one was a Mastiff, two were Labrador Retrievers, and two were of mixed breed. All had B-cell lymphoma as determined by immunophenotyping with flow cytometry [8]. Medical records and physical examination indicated that all dogs had at least stage III disease (*i*.*e*. multicentric lymphoma). At the time of diagnosis, fine needle aspirate (FNA) samples were collected from either the prescapular, submandibular, or popliteal lymph node, and a 3–5 mL blood venipuncture sample was also collected from which buffy coat (BC) leukocytes were isolated for this study. Dogs then received standard of care CHOP chemotherapy for their disease at the Mona Campbell Centre for Animal Cancer, Ontario Veterinary College, University of Guelph, Guelph, Ontario, Canada. Enrollment occurred throughout 2016–2017. The Progression Free Survival (PFS; days from diagnosis to relapse) and Overall Survival (OS) were determined; dogs were grouped into ‘poor’ outcome and ‘good’ outcome depending on median PFS of ≤ 231 or >231 days, respectively. Kaplan-Meier curves were generated and compared by log-rank analysis.

### DNA extraction and sequencing

DNA was isolated using the DNeasy Blood and Tissue kit (Qiagen, Valencia, CA), following the manufacturer’s protocol. Sample quantity and purity were assessed with a NanoDrop spectrophotometer (260/280 > 1.46, 260/230 > 0.45) and Qubit fluorometric quantitation. Samples were sent to McGill University and Génome Québec Innovation Centre, Montréal, QC, Canada, for Illumina HiSeq X Ten PE150 sequencing with qPCR and using Illumina TruSeq DNA v3 adaptors. The quality of the reads was tested and found to be acceptable; the average read quality had a Phred score of 36.

### Dataset

The dataset consisted of four DNA read files per subject: two files for BC and two files for FNA. The average number of reads in a file was 468,682,331. The average number of duplicates in a read file was 11%.

### Genome analysis

All read sets were checked for quality with *fastqc* and the reports were merged with *MultiQC* [13]. None of the read sets had any sequences that were flagged as low quality. *Trim galore* was used to improve the quality further and remove over-represented sequences. No over-represented sequences were found in any trimmed read set. The reference genome, *CanFam3*.*1*, was collected from NCBI. It was indexed with *samtools faidx* and *BWA index* [14]. The trimmed read files were then aligned to the reference genome with *BWA mem* using default parameters [14]. The aligned reads were then sorted and indexed with *samtools* [14]. *Picard* was used to mark and mask duplicates that occur as PCR artifacts. In order to have as few mismatches as possible, reads were then locally realigned using the *Genome Analysis Tool Kit (GATK)* and quality scores were recalibrated using the *VCFv4*.*1* variation file from Emsembl [15]. *Samtools mpileup* was used to calculate genotype likelihoods based on the aligned reads [14]. *VarScan* was then used to extract, call, and filter variants [16]. Variants that had a tumour variant allele frequency (VAF) >15%, a non-neoplastic sample VAF <5%, and a somatic *p*-value of <0.03 on a Fisher’s exact test were separated into high confidence mutation files [16]. These thresholds are recommended for these tools. Variants were detected in the non-neoplastic (BC) samples as well as in the tumour samples with respect to the reference genome. For this study, we were not interested in the variations present in the BC samples relative to the reference genome. Since the HapMap for dogs is not complete, any variations found in the BC samples with respect to the reference genome could represent normal, undiscovered genomic variation.

The filtered variants were then examined for genetic impact using the *Variant Effect Predictor* (*VEP*) [17]. Impact is determined via variant mapping and assignment of Sequence Ontology terms. 'High impact' terms include transcript ablation, splice acceptor variant, splice donor variant, stop gained, frameshift variant, stop lost, start lost, and transcript amplification. 'Moderate impact' terms include in-frame insertion, in-frame deletion, missense variant, and protein altering variant. The *VEP* also linked mutations to previously named mutations [17]. Human orthologues of the genes were then found using *BioMart* and re-analyzed [16]. Pseudocode steps used in this analysis are found in supporting information S1 File. The *VEP* was re-run in order to determine HGVS names for all high/moderate variants. The initial run results can be found in supporting information S2 File and the results from the additional run of the *VEP* of the variants can be found in supporting information S3 File.

## Results

**Fig 1:**
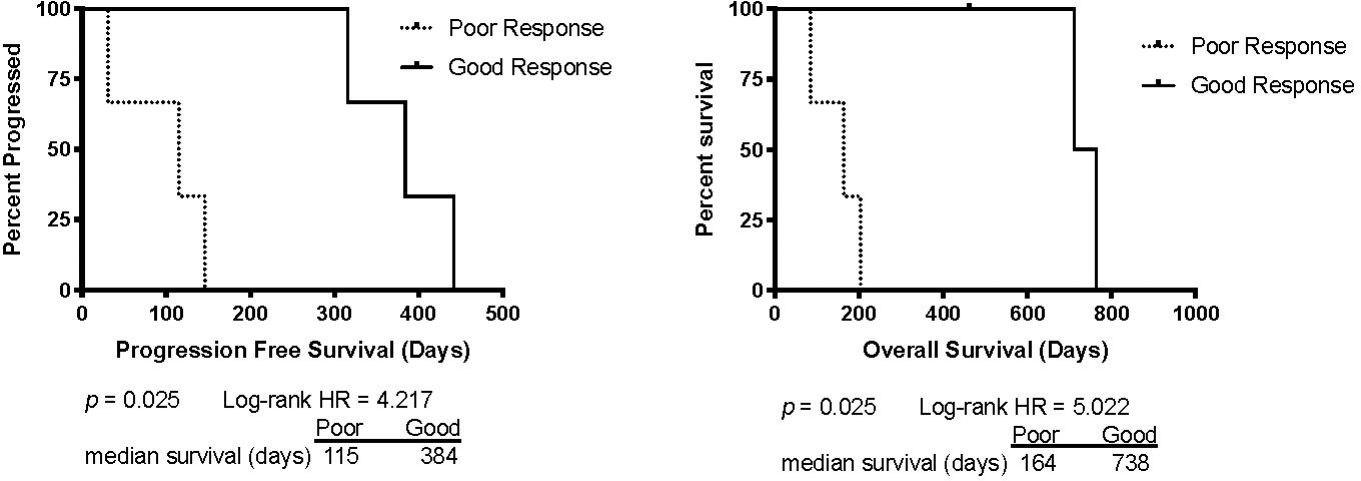
Kaplan Meier curves for Progression Free Survival (PFS) and Overall Survival (OS) for the ‘poor’ and ‘good’ outcome groups. The ‘poor’ outcome group consisted of cases with PFS ≤ 231 days and the ‘good’ outcome group consisted of cases with PFS >231 days. The one upward tick on the OS curve represents one dog that was still alive when the data were collected, so the time at last follow-up was censored. Log-rank analysis showed that there were significant differences in PFS and OS between groups.

The median PFS time for the six dogs was 231 days and the median OS was 333 days. The ‘poor’ outcome group consisted of the three patients with PFS and OS below the median, and the ‘good’ outcome group consisted of the three patients with PFS and OS above the median. In this study ‘good responder’ cases consisted of one each of stage IIIa, IVa and Vb while ‘poor responder’ cases consisted of two at stage IIIa, and one at stage Vb. There were significant differences in PFS and OS between groups, as seen in Fig 1.

A summary of variants by case and the number of high confidence variants is summarized in Table 1. The total number of high and moderate variants found in the ‘good’ and the ‘poor’ responder group was very similar. Two ‘poor responder’ cases and one ‘good’ responder case had the largest number of variants in cancer-related genes. One ‘good’ responder case had the fewest number of mutations, with none having a predicted high or moderate impact. Many of the variants were loss of heterozygosity (LOH) mutations. LOH mutations frequently occur during cancer development and the mapping of these regions has been shown to help identify novel tumour suppressor genes [18].

**Table 1:** Summary of case-specific variants.

Case Number	Total no. of variants found	Total no. variants in known genes (%)	High/moderate impact variants	Associated Table in S4 File
P1	31,966	21,690 (68%)	71	1
P2	1681	1164 (69%)	9	2
P3	32,042	23,460 (73%)	71	4
G1	17878	9983 (56%)	60	3
G2*	934	82 (9%)	0	-
G3	18,265	12,254 (67%)	78	5

‘P’ are ‘poor’ responders, and ‘G’ are ‘good’ responders.

* No genes were predicted to have a high or moderate impact in this case because all of the variants mapped to unplaced scaffolds.

**Fig 2:**
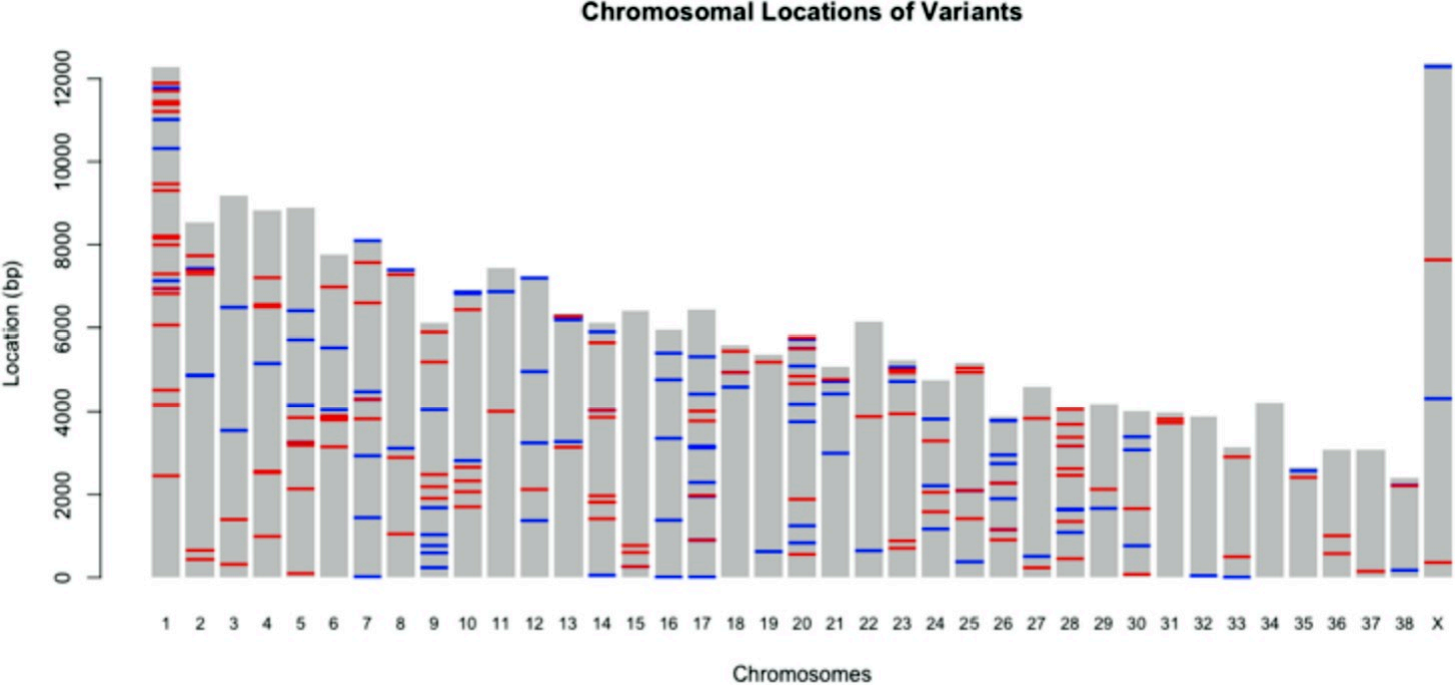
Location map of high/moderate impact genes on canine chromosomes. Variants in the ‘good’ outcome group are blue and variants in the ‘poor’ outcome group are red.

A plot of all of the high/moderate impact variants found is shown in Fig 2. Chromosome 16 and 32 only had mutations found in the ‘good’ responder group. Of these mutations, several have been previously reported in dogs (Table 2), but others are novel (Table 3). Many genes had multiple variants in different locations or cases, as summarized in Tables 4 and 5. The prevalence of the most commonly mutated genes is shown in Table 5. The gene with the most mutations, *NEFH*, had 8 total mutations, of which four occurred in a ‘poor’ outcome case and four in a ‘good’ outcome case. Information on additional genes with variants previously reported in human cancer (including NHL) is located in the supporting information S4 File. Sequence data are available from the NCBI NCI SRA, accession number PRJNA648932 (https://www.ncbi.nlm.nih.gov/sra/PRJNA648932)

**Table 2:** High/moderate impact gene mutations found in CL patients with existing, defined variant names.

Gene Name	Gene	Description	Chr	Variant Type	Existing Variation
*ANO3*	*ENSCAFG00000010152*	Anoctamin	21	SG, FS, SP	rs853126437
*BAIAP2L2*	*ENSCAFG00000001427*	BAI1 associated protein 2 like 2	10	ID	rs853028197
*CASP7*	*ENSCAFG00000011157*	Caspase 7	28	PA,SP	rs852960032
*CD3EAP*	*ENSCAFG00000004455*	CD3e molecule associated protein	1	ID	rs852110586
*DGKK*	*ENSCAFG00000016018*	Diacylglycerol kinase	X	FS	rs852072182
*DNMT3B*	*ENSCAFG00000007268*	DNA methyltransferase 3 beta	24	FS	rs850958597
*HOXA7*	*ENSCAFG00000002957*	Homeobox A7	14	ID	rs851470433
*LOXHD1*	*ENSCAFG00000017629*	Lipoxygenase homology domains 1	7	ID	rs853127792
*MAN1C1*	*ENSCAFG00000012769*	alpha-1,2-Mannosidase	2	FS, SP	rs852881997
*MEFV*	*ENSCAFG00000024473*	Pyrin innate immunity regulator	6	FS	rs852936027
*NEFH*	*ENSCAFG00000012243*	Neurofilament heavy	26	ID	rs852544148
*NOX3*	*ENSCAFG00000000589*	NADPH oxidase 3	1	FS	rs853028524
*PAX2*	*ENSCAFG00000009709*	Paired box 2	28	SA	rs851933241
*PDZRN3*	*ENSCAFG00000030541*	PDZ domain containing ring finger 3	20	II	rs852932872
*PLXND1*	*ENSCAFG00000004613*	plexin D1	20	FS, SP	rs852296177
*PTCH1*	*ENSCAFG00000001246*	Patched 1	1	SD, FS	rs851061836
*RBM48*	*ENSCAFG00000001980*	RNA binding motif protein 48	14	ID	rs850548548
*SETX*	*ENSCAFG00000019897*	Senataxin	9	ID	rs852619957
*ST6GALNAC5*	*ENSCAFG00000020386*	ST6 N-acetylgalactosaminide alpha-2,6-sialyltransferase 5	6	ID	rs850967165
*TMCC2*	*ENSCAFG00000010030*	Transmembrane and coiled-coil domain family 2	38	ID	rs851329311
*UBXN6*	*ENSCAFG00000019059*	UBX domain protein 6	20	FS, SP	rs852129638
*-*	*ENSCAFG00000029562*	-	1	FS	rs851972674
*-*	*ENSCAFG00000030746*	-	1	FS	rs851216535
*-*	*ENSCAFG00000007230*	-	1	FS	rs850663404
*-*	*ENSCAFG00000001689*	-	10	FS	rs850767066
*-*	*ENSCAFG00000031399*	-	13	FS	rs852882764
*-*	*ENSCAFG00000029044*	-	13	FS	rs852592769
*-*	*ENSCAFG00000003769*	-	14	ID	rs852183254
*-*	*ENSCAFG00000030817*	-	16	FS	rs852252464
*-*	*ENSCAFG00000007370*	-	17	FS	rs852027546
*-*	*ENSCAFG00000008121*	-	17	FS, SP, IN	rs853022647
*-*	*ENSCAFG00000005292*	-	22	FS	rs852830764
*-*	*ENSCAFG00000025335*	-	24	FS	rs852293039
*-*	*ENSCAFG00000024806*	-	25	SA, FS	rs853028527
*-*	*ENSCAFG00000025297*	-	25	ID	rs852801980
*-*	*ENSCAFG00000031273*	-	26	FS	rs851011185
*-*	*ENSCAFG00000039374*	-	28	SD, NCT	rs850917564
*-*	*ENSCAFG00000012412*	-	28	FS	rs850985714
*-*	*ENSCAFG00000029487*	-	30	ID	rs852745913
*-*	*ENSCAFG00000031064*	-	32	II	rs850972929
*-*	*ENSCAFG00000024272*	-	35	ID	rs851176735
*-*	*ENSCAFG00000029384*	-	7	ID	rs850875903
*-*	*ENSCAFG00000024792*	-	9	ID	rs852788717
*-*	*ENSCAFG00000024792*	-	9	II	rs851118190

Specific mutation information can be found in Tables in S4 File. Not all variants have been previously linked to CL. SG–Stop gained, FS—Frameshift, SP—Splice region, ID–in-frame deletion, PA–protein altering, SA—Splice acceptor, II–in-frame insertion, SD -Splice donor, IN–intron, NCT–non-coding transcript exon.

**Table 3:** High/moderate impact genes with variants not previously identified in canine lymphoma.

Gene Name	Description	Chr	Variant Type
*ALAS1*	5'-aminolevulinate synthase 1	20	In-frame Deletion
*ANGPTL6*	Angiopoietin like 6	20	In-frame Insertion
*ATPAF2*	ATP synthase mitochondrial F1 complex assembly factor 2	5	In-frame Insertion
*ATRNL1*	Attractin like 1	28	In-frame Insertion
*C10orf62*	Chromosome 10 open reading frame 62	28	Splice Acceptor, Coding
*CCSAP*	Zinc finger protein 384	4	In-frame Deletion
*CSPP1*	Centrosome and spindle pole associated protein 1	29	Frameshift
*CHST14*	Carbohydrate sulfotransferase	30	Frameshift
*COX18*	Cytochrome c oxidase assembly factor	13	In-frame Insertion
*DNTB*	Dystrobrevin beta	17	Frameshift
*EFNB3*	Early B-cell factor 1	4	Frameshift
*FANCF*	Fanconi anemia complementation group F	21	In-frame Deletion
*FBXO34*	F-box protein 34	8	Frameshift
*FOXB2*	Forkhead box B2	1	Frameshift
*GPC1*	Glypican 1	25	Frameshift
*GPRASP1*	G protein-coupled receptor associated sorting protein 1	X	Frameshift
*HCN1*	Hyperpolarization activated cyclic nucleotide gated potassium channel 1	4	In-frame Insertion, Splice Region
*HSPG2*	Heparan sulfate proteoglycan 2	2	Frameshift
*KCNH3*	Potassium voltage-gated channel subfamily H member 3	27	Frameshift
*KIAA1614*	Uncharacterized protein	7	In-frame Insertion
*KIF2A*	Kinesin-like protein	2	Frameshift
*KRT10*	Keratin, type 1 cytoskeletal 10: epidermal barrier on plantar skin	9	In-frame Deletion
*LRP3*	LDL receptor related protein 3	1	Frameshift, Splice Region
*LRRN4*	Leucine rich repeat neuronal 4	24	In-frame Insertion
*MAN1C1*	Alpha-1,2-Mannosidase	2	Frameshift, Splice
*MUC20*	Mucin 20, cell surface associated	33	Frameshift
*MKI67*	Marker of proliferation Ki-67	28	In-frame Insertion
*MORN1*	MORN repeat containing 1	5	Frameshift
*MRPS30*	Mitochondrial ribosomal protein S30	4	In-frame Insertion
*NKX1-2*	NK1 homeobox 2	28	In-frame Insertion
*PLPP6*	Phospholipid phosphatase 6	1	In-frame Insertion
*PRRT3*	Proline rich transmembrane protein 3	20	In-frame Insertion
*ENSCAFG00000030179 (RFK orthologue)*	Riboflavin kinase	1	Frameshift
*RFX1*	Regulatory factor x1	20	Frameshift
*RIOX2*	Ribosomal oxygenase 2	33	In-frame Insertion
*RYR1*	Ryanodine receptor 1	1	Frameshift
*SH3BGRL3*	SH3 domain binding glutamate rich protein like 3	2	Frameshift
*SYTL1*	Synaptotagmin like 1	2	Frameshift
*TENM3*	Teneurin transmembrane protein 3	16	Frameshift, In-frame Insertion
*TMEM178A*	Transmembrane protein 178A	17	Frameshift, Splice Region
*TNP2*	Nuclear transition protein 2	6	Frameshift
*TRABD2A*	TraB domain containing 2A	17	Frameshift
*TSPYL1*	TSPY like 1	12	Frameshift
*TTC21A*	Tetratricopeptide repeat domain 21a	23	Frameshift
*WFDC10A*	WAP four-disulfide core domain 10A	24	Frameshift
*ZCCHC3*	Zinc-finger CCHC-type containing 3	24	In-frame Deletion

Variants in these genes have not been previously reported in CL studies. Specific mutation information can be found in Tables in S4 File.

**Table 4:** Common variants in subsets of cases.

Case Numbers	Total N+T	Total T only	Total Common Variants	Total No. Genes Defined	No. Moderate or High Impact Variants
P1 & G1	100	63	163	68	0
P1 & P2	28	22	50	6	1
P1 & P3	847	137	984	768	4
P1 & G3	147	92	239	154	0
G1 & P2	26	0	26	8	0
G1 & P3	105	62	167	90	1
G1 & G3	87	40	127	60	0
P2 & P3	33	25	58	18	0
P2 & G3	46	25	71	24	0
P3 & G3	200	126	326	279	2
P1, P3 & G1	21	0	21	4	0
P1, G1 & G3	22	0	22	3	0
P1, P2 & G3	21	0	21	0	0
P1, P3 & G3	28	24	52	5	0

N+T represents a variant present in the tumour and as a heterozygous allele in the buffy coat. T represents enrichment in the corresponding gene in the tumor sample. ‘P’ represents a patient in the ‘poor responder’ group and ‘G’ represents a patient in the ‘good responder’ group.

**Table 5:** Most commonly mutated genes and their human orthologues.

# Occur.	Dog gene ID	Gene name (Human gene)	Human gene ID
8	*ENSCAFG00000012243*	*NEFH*	ENSG00000100285
6	*ENSCAFG00000008123*	*TENM3*	ENSG00000218336
6	*ENSCAFG00000015252*	*NME7*	ENSG00000143156
6	ENSCAFG00000030928		
4	ENSCAFG00000007370	*(IGKV4-1)*	ENSG00000211598
4	ENSCAFG00000029044		
4	ENSCAFG00000032540		
3	ENSCAFG00000020296		
3	ENSCAFG00000029275		
3	ENSCAFG00000029320		
3	ENSCAFG00000030512		
3	ENSCAFG00000031464		
2	ENSCAFG00000001138		
2	ENSCAFG00000001427	*BAIAP2L2*	ENSG00000128298
2	ENSCAFG00000002957	*HOXA7*	ENSG00000122592
2	ENSCAFG00000002962	*COX18*	ENSG00000163626
2	ENSCAFG00000003432	*CTTNBP2*	ENSG00000077063
2	ENSCAFG00000004584	*TSC22D1*	ENSG00000102804
2	ENSCAFG00000005530	*ZFP36*	ENSG00000128016
2	ENSCAFG00000005547	*RBFOX3*	ENSG00000167281
2	ENSCAFG00000005896	*RYR1*	ENSG00000196218
2	ENSCAFG00000007268	*DNMT3B*	ENSG00000088305
2	ENSCAFG00000008571	*KCNH3*	ENSG00000135519
2	ENSCAFG00000011725	*ATRNL1*	ENSG00000107518
2	ENSCAFG00000012826	*KIAA1614*	ENSG00000135835
2	ENSCAFG00000014740	*ZNF384*	ENSG00000126746
2	ENSCAFG00000014773	*HSPG2*	ENSG00000142798
2	ENSCAFG00000017316		
2	ENSCAFG00000017629	*LOXHD1*	ENSG00000167210
2	ENSCAFG00000018508	*HCN1*	ENSG00000164588
2	ENSCAFG00000019353	*(PRSS27)*	ENSG00000172382
2	ENSCAFG00000020386	*ST6GALNAC5*	ENSG00000117069
2	ENSCAFG00000024792	*(CD300C/CD300A)*	ENSG00000167850*/*ENSG00000167851
2		*(CD300A)*	ENSG00000167851
2	ENSCAFG00000028602		
2	ENSCAFG00000029209		
2	ENSCAFG00000029370		
2	ENSCAFG00000029963		
2	ENSCAFG00000030179	*(RFK)*	ENSG00000135002
2	ENSCAFG00000030746		
2	ENSCAFG00000030849		
2	ENSCAFG00000030864		
2	ENSCAFG00000031089		

If the canine gene name is missing, the human gene name has been added in brackets.

**Fig 3:**
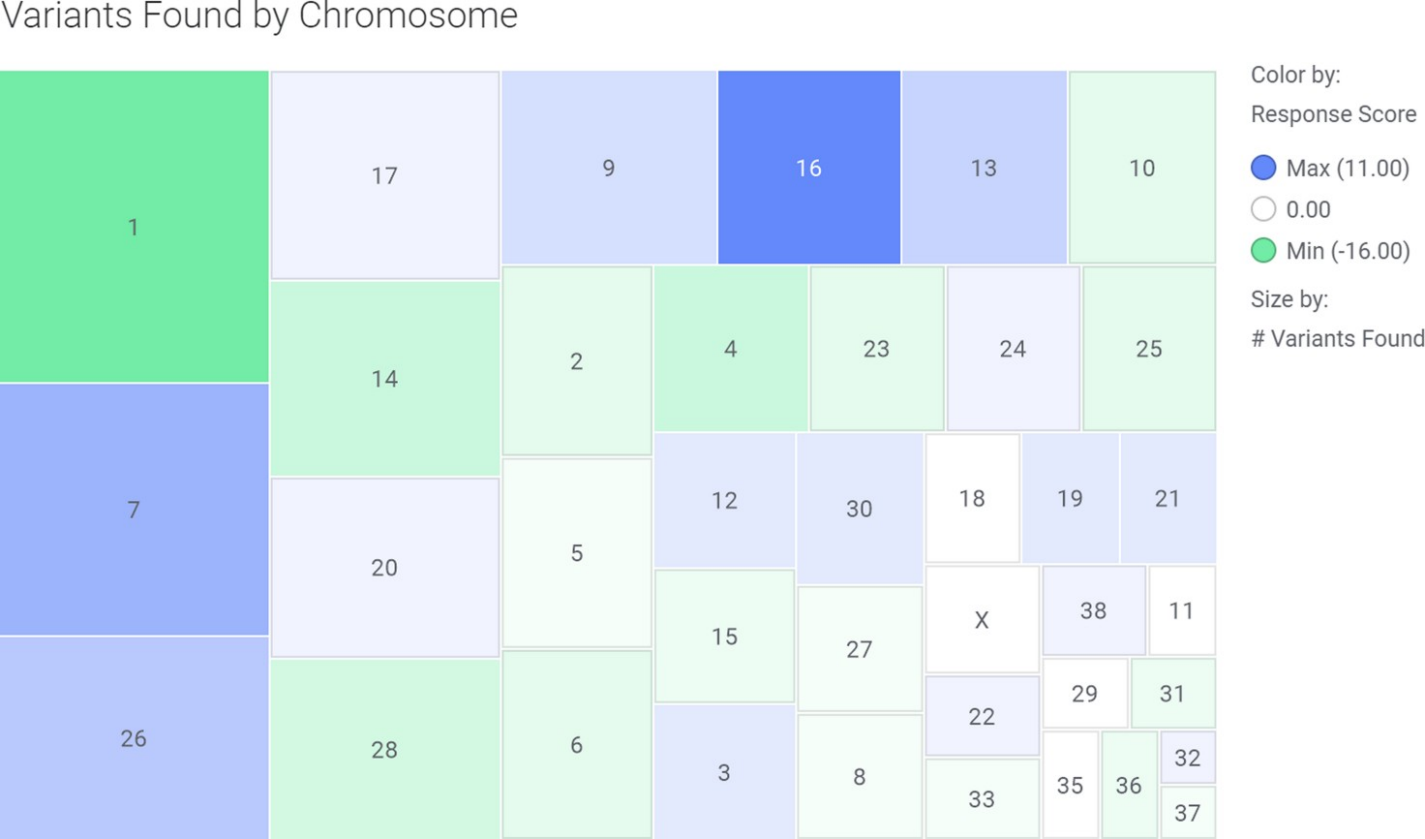
Heat map of response score in high/moderate variants by chromosome. Variants were given a response score of -1 if they occurred in a ‘poor’ responder and a response score of +1 if they occurred in a ‘good’ responder. All offset scores for high/moderate variants were totaled for each chromosome whereby a positive total score represents more variants found in ‘good’ responders than ‘poor’ responders and vice versa. The response scores were plotted in a heat map where the size and order of the chromosome’s box is determined by the total number of high/moderate variants found within the chromosome and the colour represents the total response score for the chromosome (green = negative score and blue = positive score). The chromosome with the lowest response score was chromosome 1 with -16 and the chromosome with the highest response score was chromosome 16 with 11.

A heat map of response scores by chromosome is shown in Fig 3. The heat map shows both the relative amount of high/moderate variants found in each chromosome as well as the proportion of variants found in ‘good’ vs ‘poor’ responders. The most commonly mutated chromosomes are 1 and 7. The least commonly mutated chromosomes are 32 and 37. The chromosome with the highest proportion of ‘good’ responder variants is 16 with 11 more variants found in ‘good’ responders than variants found in ‘poor’ responders. The chromosome with the highest proportion of ‘poor’ responder variants is chromosome 1 with 16 more ‘poor’ responder variants than ‘good’ responder variants. Chromosomes 11, 18, 29, 35, and X all had equal numbers of variants in both ‘good’ and ‘poor’ responders.

**Fig 4:**
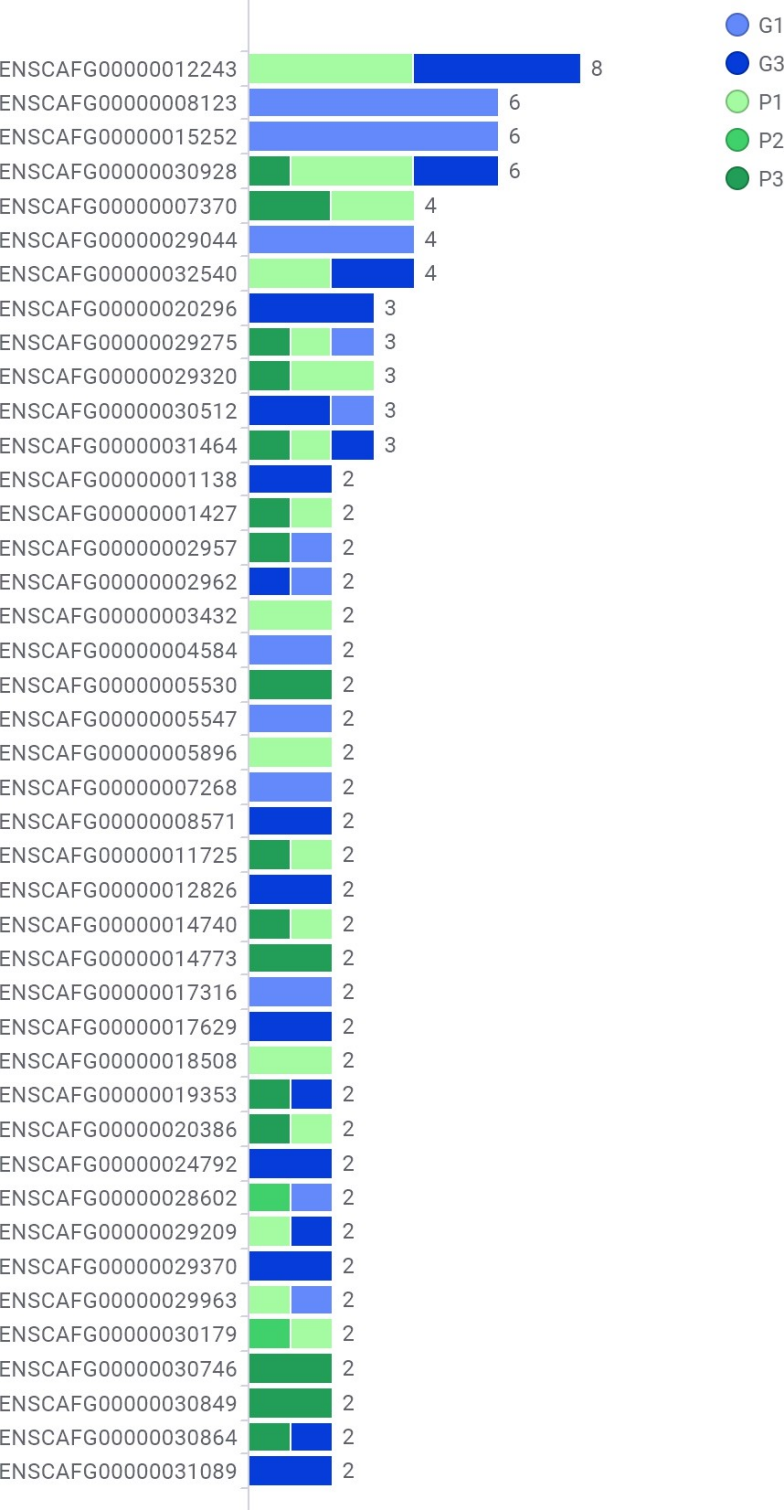
Common variants by case. Genes with variants with a prevalence greater than one mapped by case. Each horizontal bar represents a variant. Coloured bar segments represent cases. ‘P’ represents a patient in the ‘poor responder’ group (green bar segments) and ‘G’ represents a patient in the ‘good responder’ group (blue bar segments). ‘P’ represents a patient in the ‘poor responder’ group and ‘G’ represents a patient in the ‘good responder’ group.

Fig 4 shows a schematic of variant gene by prevalence and case. The three most commonly mutated genes are *ENSCAFG00000012243* (*NEFH*), *ENSCAFG00000008123* (*TENM3*), and *ENSCAFG00000015252* (*NME7*).

## Discussion

There were 11 genes with variants found in both ‘good’ and ‘poor’ responders, but only two of these variants were in genes named in the dog: *NEFH* and *HOXA7*. One unnamed gene found in both ‘good’ and ‘poor’ responders, *ENSCAFG00000019353*, has a named human orthologue, serine protease PRSS27, while the rest of the common genes are not yet named. BLAST search results of the canine sequences for these unnamed genes against the human database are included in supporting information S5 File.

We discovered eight different mutations in *NEFH*: four in ‘poor’ responders and four in ‘good’ responders. Of the eight mutations, two were predicted to have high or moderate impact. Found in both ‘good’ and ‘poor’ responders, these were 24 base deletions and included parts of both exon 4 and intron 4. These mutations, which impact the edge of an exon, have potential to affect *NEFH* splicing, but since they are the same length and occur in the same region, these mutations are probably not relevant to the pathogenesis of lymphoma. For *HOXA7*, the same mutation, rs851470433, was found in both the ‘good’ and ‘poor’ responder groups. This three base pair deletion occurred in a simple CGG repeat region. Again, it is unlikely that this mutation affects CL progression, as it was found in both ‘poor’ and ‘good’ responder groups.

There were seven variants found in at least two ‘poor’ responders and not in any ‘good’ responders: ENSCAFG00000011725 *(ATRNL1)*, ENSCAFG00000001427 *(BAIAP2L2)*, ENSCAFG00000014740 *(ZNF384)*, ENSCAFG00000020386 *(ST6GALNAC5)*, ENSCAFG00000030179 (human ortholog: riboflavin kinase RFK), ENSCAFG00000029320, and ENSCAFG00000007370 (human orthologue: IGKV4-1) [19]. ENSCAFG00000011725 (*ATRNL1*), which codes for attractin-like protein 1, had a 12 base pair long variant exon 1/29. In *ENSCAFG00000001427* (*BAIAP2L2*), a six base pair, in-frame deletion was located within a CpG island in exon 12 of 16 [20]. This *BAIAP2L2* mutation has been previously found in dogs and named rs853028197 [21]. The mutation rs853028197 is a 6 base deletion in the middle part of the gene. *BAIAP2L2* (aka *FLJ22582*) as well as rs853028197 are uncharacterized in both dogs and humans, so the effect of this mutation is difficult to predict. *BAIAP2L2* is over-expressed in human liver tumour tissues due to hypo-methylation, but as yet, deletions in this gene are not reported in canine cancer [22]. This small in-frame deletion likely does not have a significant effect on protein function, however, there is strong evidence that BAIAP2L1 promotes cell proliferation and inhibits apoptosis [23] and BAIAP2L2 may have a similar role in dog and human lymphoma.

In *ENSCAFG00000014740 (ZNF384)*, a non-frameshift deletion of 30 bases was discovered in exon 11 in a (CCCCAG)_n_ simple repeat region and a non-frameshift insertion of 18 bases was found in exon 11 of 11 [24]. *ZNF384* is a part of a fusion gene pair found in B-cell precursor acute lymphoblastic leukemia (ALL) [25]. It is unknown whether this mutation would have any effect on the ability of *ZNF384* to form a fusion gene. Two non-frameshift deletions were also found in *ENSCAFG00000020386 (ST6GALNAC5)* of length six and three base pairs. Both mutations occur in CpG islands in exon 1 of 4, and the latter has been previously found and named rs850967165 [20]. rs850967165 is uncharacterized and results in removal of a glutamine residue at the 46^th^ of 334 amino acids. The deletion occurs at the start of a (CTG)_n_ simple repeat region [24]. CpG islands are usually associated with promoters [20], and mutations here can affect expression levels of the associated protein [26]. *ST6GALNAC5* is overexpressed in breast cancer cells and has been strongly linked to brain metastases [27]. Further investigation into this mutation could help determine its effect on canine lymphoma progression. ENSCAFG00000030179 has a human orthologue of *RFK* (riboflavin kinase). RFK catalyzes the phosphorylation of riboflavin in FAD synthesis and is involved in ROS metabolic processes and apoptotic processes [28]. There were two instances of an insertion of a G in exon 1 of 4, in a GpG island [20].

Two deletions in ENSCAFG00000007370 were only found in ‘poor’ responder patients: a six base pair, in-frame deletion and a single base pair, frameshift insertion. Both mutations were in exon 2, and the single base pair insertion has been previously named rs852027546. The uncharacterized rs852027546 mutation causes a frameshift change of 42 amino acids starting with a glycine to glutamic acid residue in the Ig-like domain of ENSCAFG00000007370. A mutation of this severity has the potential to completely disrupt protein function. *IGKV4-1* is often mutated and rearranged before being expressed as the kappa light chain in immunoglobulins which go on to bind antigens, causing clonal expansion and a specific immune attack [29]. We postulate that a severely truncated IGKV4-1 component could result in an immunoglobulin without a light chain, without a functional light chain, or no immunoglobulin at all. If this mutation inhibited immunoglobulin production, it could free the cells from a primary, obligatory, and high-energy task, leaving more resources to help them survive.

There are two genes with changes found in at least two ‘good’ responders and not in any ‘poor’ responders: *COX18*, and *ENSCAFG00000030512*. COX18 is involved in the insertion of cytochrome c oxidase 2 into the inner mitochondrial membrane, which is involved in cellular metabolism [30]. Mutations in this gene are considered to be generally incompatible with cell survival and occur very infrequently [30, 31]. In our study, mutations in *COX18* occurred frequently, but only in lymphomas in the ‘good’ responder group. It may therefore be helpful to evaluate whether targeting this gene in combination with CHOP therapy will affect outcomes. In the dogs in this study, *COX18* had a frameshift deletion and a non-frameshift deletion that were both located in CpG islands in exon 1 of 6 [20]. A frameshift mutation in the first exon could disrupt the translation of a majority of the protein. Since *COX18* codes for a widely used metabolic enzyme, absence thereof might severely impact tumor cell growth and survival. For ENSCAFG00000030512, there were frameshift deletions of TT and T, respectively, both in a simple tandem repeat region of TTTC in exon 2 of 2 [32]. Tandem repeat regions can be highly variable, but large insertions are more likely to be disease-related than short deletions within such regions [33], thus, this mutation in ENSCAFG00000030512 may not have a functional impact on CL progression.

Many mutations found in this study are involved in processes, such as cellular differentiation, growth, survival, and transformation, which have the potential to trigger or intensify cellular dysregulation. Variants in *SH2B3*, *PAX2*, *HOXB3*, *GLS*, and *ZNF503* were only found in individual ‘poor’ responder cases. *SH2B3* (SH2B adaptor protein 3), is poorly studied in dogs, but in humans it regulates the generation of certain lymphocytes and expansion of hematopoietic stem cells, and may affect inflammation in peripheral lymphoid tissues [34]. In humans, *SH2B3* has been called a recessive tumour suppressor gene whose mutation may lead to ALL [35]. A frameshift mutation could result in the loss of the tumour suppressing functions of *SH2B3* and may contribute to a faster progressing disease. *PAX2* is usually only expressed during embryogenesis in humans, but expression of *PAX2* has been found in cell lines of human lymphoma and other cancer types, where it promotes cell growth and survival [36, 37]. If the mutated *PAX2* mRNA contains introns or is missing exons, it will probably have altered functionality, but if the single mutation does not affect splicing, *PAX2* expression could cause a more aggressive CL presentation. *HOXB3* is a part of the Hox family of genes. Dysregulation of the Hox family is found in a majority of acute myeloid leukemia cases and many ALL cases [38]. In our case, a three base pair insertion is not likely to have a significant effect on the protein’s function.

The glutamine metabolic pathway is important for cancer growth and GLS has been studied in both human and canine mammary tumours [39]. There is an association between GLS1 (an isoform of GLS) expression, malignancy, and tumour type of canine mammary tumours [39]. Our finding of an in-frame insertion of six bases in *GLS* may allow the protein to function normally in the glutamine metabolic pathway. In *ZNF503*, we found a frameshift deletion. Previously, a focal deletion was found in zinc finger protein 503 in dogs with mammary tumours [40]. These two mutations could both result in non-functional proteins and may have an effect on CL progression. Since ZNF503 is not characterized in dogs, investigating this effect would require functional analysis.

Variants found only in ‘good’ responder cases are also of interest; *TP53*, *SETD2*, and *PTCH1* were all affected. These mutations may impact response to therapy and/or be involved in evolution of tumours that are inherently less aggressive in behaviour. TP53 is involved in cell cycle arrest, DNA damage response, and apoptotic process and has been reported as a possible tumour initiation gene for NHL [12]. *TP53* deletions occur in up to 20% of human diffuse large B-cell lymphomas in humans and negatively impact survival [41]. TP53 deletions and point mutation incidence is reported to range from 14–37.5% of cases in canine lymphoma, although the impact on outcome and resistance to therapy is variable. [42–45]. The SET domain containing 2 gene (*SETD2*) was previously found to be mutated in CL patients [10]. *SETD2* is also recurrently mutated in whole-exome sequenced canine osteosarcomas, T-cell lymphomas, and diffuse large B-cell lymphomas [46–49]. A frameshift mutation in this gene may cause a non-functional protein that cannot suppress tumour formation [50]. PTCH1 is a receptor in the Hedgehog signaling pathway that is involved with the self-renewal of hematopoietic stem cells in dogs as well as humans [51, 52]. We found a frameshift mutation in *PTCH1* that could lead to lost functionality. Lower levels of PTCH1 have been linked to a higher risk of metastasis in human colorectal cancer [52].

Variants in *PPP2CB* and *CD3EAP* were also found in only ‘good’ responders. *PPP2CB* encodes a serine/threonine-protein phosphatase (PP2A), which is involved in apoptosis and cell cycle regulation [53]. *PP2A* is a well-conserved tumour suppressor and a frameshift mutation in *PPP2CB* could cause *PP2A* to lose functionality and cause disordered cell proliferation and a more aggressive cancer progression [54]. *CD3EAP*, CD3E molecule associated protein, is involved in the regulation of apoptosis and transcription [55]. CD3EAP interacts with nuclear factor-kappa-B (NFKB1) and cellular tumor antigen p53 (TP53) proteins and is associated with ‘poor’ outcome for multiple myeloma patients [55]. However, the in-frame deletion found here would probably not have a significant effect on function. Although these findings are apparently not consistent with a good prognosis, further studies may reveal deeper understanding of the effect of *PPP2CB* and *CD3EAP* in CL progression.

Most genomic studies in dogs tend to study a small section of DNA that may be linked to a certain phenotype. Our study takes a different approach, as there was no specific genetic target selected for analysis. We are also the first to use whole genome sequencing (WGS) as an exploratory method to find any and all possible genetic contributors to canine B-cell lymphoma. A strength of WGS is that prior knowledge does not limit the scope of the analysis; previously unrelated genes can be linked to new diseases. Thus, for CL, where the mechanism of disease progression is far from known, an approach like WGS can reveal new and relevant genomic mutations.

A limitation of this study was its small sample size. A larger sample size would give more weight to commonly found variants and the strength of variant assignment to ‘good’ and ‘poor’ response groups. The low percentage of defined genes associated with the variants found in our study is also a problem for the further analysis of lymphoma associated variants. With an average of only 56% of variants having an associated defined gene, there is a large possibility that an important variant was missed with this analysis. Furthermore, there is lack of knowledge of canine proteins, their interactions, and their associated GO terms, requiring the use of human orthologues for parts of the analysis. There does not yet exist a canine equivalent of the human HapMap. Hence, some variants identified here may represent normal variation. It is also important to note that the non-neoplastic tissue samples were taken from the buffy coat of patients so it is possible that there was a small number of circulating lymphoma cells. None of our cases were evaluated by bone marrow aspirate because clinically relevant cytopenia or atypical circulating cells were not observed. However, the presence of circulating neoplastic cells could affect the identification and interpretation of variants in our study. In particular, it is possible that we are underestimating the number of disease-related variants (*i*.*e*. those found in neoplastic samples and not in somatic DNA).

The most promising variants found in our study were *in ST6GALNAC5*, ENSCAFG00000007370, *PPP2CB*, *TP53*, *SH2B3*, *ZNF503*, *SETD2*, and *COX18*. As knowledge continues to expand regarding CL genetics, the goals are to improve the prognosis of affected dogs and to potentially apply findings to humans. It is worth noting that subtypes in CL are not defined with the same criteria as human NHL [3]. Defining subtypes of CL more accurately may thus help with clustering of genome-wide analysis data [56]. It is also worth considering that genetic causes of CL may not be the same in every breed, and different breeds may represent distinct genetic units [10]. Thus, it may be beneficial for reads to be mapped to a reference genome of the same breed, rather than only the purebred Boxer represented in the *CanFam3*.*1* reference sequence. A variant file for each breed, created with respect to the reference sequence would allow recalibration of read alignments and could filter out unimportant genomic changes. It follows that variants found in multiple breeds have more importance in the disease processes of CL, which could help to identify better candidate mutations for CL.

## Supporting information

S1 File Pseudocode steps for bioinformatic analysis. (PDF)

S2 File Initial, combined results from the variant effect predictor. (XLSX)

S3 File Results from the additional run of the variant effect predictor (includes HGVS naming). (XLSX)

S4 File Supplementary information on other gene variants identified in this study. (PDF)

S5 File Results of BLAST analysis for unknown genes. (XLSX)
