# Supplementary material for: Whole genome sequencing analysis of high confidence variants of B-cell lymphoma in Canis familiaris
Source: PLoS One. 2020 Aug 28;15(8):e0238183. doi: 10.1371/journal.pone.0238183 (PMC7454977; doi:10.1371/journal.pone.0238183)
Supplement: S1 File — (PDF) [file pone.0238183.s001.pdf]

S1 File: Pseudocode steps

1. Wget reference genome from ncbi (CanFam3.1)
2. Samtools faidx reference genome
3. Bwa index reference genome
4. Wget raw reads from genomequebec.mcgill
5. Factqc –noextract raw reads
6. Multiqc all factqc reports
7. Trim galore –paired
8. Fastqc –noextract trimmed reads
9. Bwa mem –R trimmed reads
10. Samtools sort aligned reads
11. Samtools index sorted reads
12. Samtools dict reference genome
13. GATK RealinerTargetCreator
14. GATK IndelRealigner
15. GATK BaseRecalibrator
16. GATK PrintReads
17. Picard MarkDuplicates
18. Samtools mpileup
19. VarScan somatic mode
20. Vcf Intersecting and Comparing
21. Variant Effect Predictor
22. Human orthologue analysis
