# Supplementary material for: Whole genome sequencing analysis of high confidence variants of B-cell lymphoma in Canis familiaris
Source: PLoS One. 2020 Aug 28;15(8):e0238183. doi: 10.1371/journal.pone.0238183 (PMC7454977; doi:10.1371/journal.pone.0238183)
Supplement: S4 File — (PDF) [file pone.0238183.s004.pdf]

S4 File: Supplementary Information

Many of the genes with variants found in our study have been previously identified as having tumour suppressor functions in humans. DCHS2 has been reported as a tumour suppressor gene that truncates fusion genes to cause loss of function [1]. PLK5 and DNMT3B have been identified as tumour suppressor genes in different cell lines and cancers [2-4]. ZFP36, also known as tristetraprolin (TTP), has tumour suppressor functions [5]. PLA2R1 is a receptor that has been shown to cause several tumour suppressive responses including senescence, apoptosis and inhibition of transformation [6]. The PPP2R1B gene product may suppress tumour development, so a frameshift mutation could cause the tumour suppression to fail and actually cause the cancer to be more aggressive [7]. KAT6B has been shown to have tumour suppressor function and has recurrent rearrangements in leukemia, sarcomas and midline carcinomas [8]. GATA6 has a tumour-suppressive function [9]. The high and moderate impact variations found in these genes are predicted to largely affect their functions. Due to the tumour suppressing functions of these genes, they may have had a large role in the development or progression of lymphoma in the cases included in this study.

There are existing links between human lymphomas and many of the genes found to be mutated in this study. Regional mutations have been found in SEMA6B in T-Cell lymphoma [10]. Human CIC mediates RTK-dependent responses that are linked to cell proliferation and cancer [11]. Simon-Carrasco et al. and Tan et al. have induced T-cell lymphoblastic lymphoma through the inactivation of CIC in adult mice [12, 13]. MLLT3 has been shown to fuse with MLL (KMT2A) in lymphoblastic lymphoma [14]. Genetic mutations in relation to lymphoma have not been previously defined for these genes.

Many of the other mutated genes found in this study have human orthologues with links to cancers other than lymphoma. ADAM22 is a potential pro-metastatic gene in endocrine resistant cell lines in humans [15]. ADAM22 promotes migration and de-differentiation, therefore, increasing survival and colonizing abilities of metastases [15]. ADAM22 is being studied as a therapeutic target for breast cancer in humans [15]. BAIAP2L2 has been discovered to be hypo-methylated in human liver tumour tissues [16]. CTTNBP2 is involved in aggressive prostate cancer in humans [17]. CTTNBP2 is involved in the blockage of genes on chromosome 7 that are differentially methylated in prostate cancer cells [17]. DPP4A is the human orthologue for an unnamed gene. It is involved in self-renewal and pluripotency sustainability in embryonic stem cells [18]. DPP4A is often re-expressed in malignant tumours [18]. It is considered a new pluripotency-related oncogene [18]. DPP4A is also expressed in bladder, prostate, hepatic, and colon cancers and cancerous cell lines [19]. KRT76 is downregulated in human oral cancers, but had been determined to be an early event that does not drive cancer development [20]. Eight variants have been found in MRPS30 in human breast cancer patients [21]. NEFH is involved in primary esophageal squamous cell carcinomas (SCC) [22]. In knockdown experiments, there is accelerated cell growth and increased tumorigenicity [22]. NOX3 is a NADPH oxidase enzyme that generates reactive oxygen species (ROS). The NOX family of enzymes are involved in a variety of normal physiological processes. An imbalance in the NOX family of enzymes has potential for acute and chronic disease due to genomic instability [23]. For example, high levels of ROS can increase risk for cancer development [23]. NOX1, NOX4, and NOX5 are the most studied NOX family enzymes that are linked to human cancer [23]. NOX3 is less well understood [23]. NOX3 expression has been discovered to increase after heavy ion radiation and contributes to radiation-induced death [24]. PELP1 is an estrogen receptor co-activator, a co-

regulator for many transcription factors, involved in chromatin remodeling complexes, and has cytoplasmic signaling functions [25]. PELP1 increases proliferation and metastasis in breast, endometrial, ovarian, colon, salivary gland and prostate cancers, as well as astrocytic brain tumours. RBM48 is an essential nuclear protein [26]. In humans, it interacts with ARMC7 and is commonly activated in major cancers [26]. SH2B3 has been shown to have anti-proliferative effects in hematopoietic cells, but when overexpressed in ovarian cancer cell lines, SH2B3 can cause cells to resist death in certain conditions [27]. ST6GALNAC5 has been shown to mediate breast cancer metastasis to the brain by decreasing interactions between cancer cells and human blood brain barrier cells [28]. ZNF384 is a part of fusion genes that have been found in B-cell precursor ALL [29]. The APOL family consists of six genes that have been linked to many diseases in humans, including pancreatic, breast, and liver cancer. APOL1 overexpression induces autophagic cell death in cancer cells and mutations in APOL1 may cause impairment of autophagy, which has been linked to pathogenesis of different cancer types [30]. A pancreatic cancer case–control study discovered an association between a SNP within the APOL3 locus and pancreatic cancer risk [31]. HOXA7 protein was absent in normal surface epithelium but appeared in metaplastic regions [32]. HOXA7 promoted metastasis of HCC patients and stimulated proliferation in breast cancer patients [32, 33]. TSC22D1 as novel candidate genes for spontaneous pulmonary adenomas [34]. RBFOX3 has been shown to promotes tumour growth and progression [35]. PSMA1 has been reported as a colon cancer marker because it is only found in cancerous tissues [36]. Evidence found that NME7 deregulation may contribute to carcinogenesis due to its role in maintaining pluripotency and stem cell renewal [37]. CNTNAP3 was found to be upregulated in human breast cancer-associated fibroblasts and CNTNAP3B was associated with poor prognosis in lung adenocarcinomas [38]. PANK1 deletions were found in

5.9% of prostate cancer cases in a study by Kluth [39]. SNPs in PLCH1 were strongly associated with SCC [40]. Expression of PLA2R1 decreases in numerous cancers, and it has been shown to reduce the expression of various oncogenes. A CASP7 polymorphism was associated with cervical cancer risk [41]. PPP2R1B has been found to have variations in low percentages of primary lung tumours, lung tumour derived cell lines, and primary colon tumours [7]. A deletion mutation has been found that truncates the encoded protein, making it unable to bind to the catalytic subunit of the protein phosphatase 2A holoenzyme. SNPs in PGLYRP2 are statistically significantly associated with esophageal SCC [42]. MEFV has shown potential to contribute to the cause of ALL [43]. WHRN (whirlin) was found to be up-regulated in colorectal cancer tissues and associated with tumour progression [44]. DNMT3A interacts directly and localize to promoters silenced in cancer cells and has been associated with more aggressive tumour behavior [45]. A SNP in EDEM1 was found in breast cancer patients [46]. CTSV has been linked to breast cancer metastasis [47]. Deletions were detected in EBF1 in patients with ALL [48]. LOXHD1 was significantly mutated in ampullary carcinomas [49].

The specific mutations found in each case have been defined in the Supplementary Tables. Supplementary Table 1, 2, 3, 4, and 5 outline the mutations found in case P1, P2, P3, G1 and G3, respectively.

1. Dhanasekaran, S.M., et al., *Transcriptome meta-analysis of lung cancer reveals recurrent aberrations in NRG1 and Hippo pathway genes.* Nat Commun, 2014. **5**: p. 5893.
2. de Carcer, G., et al., *Plk5, a polo box domain-only protein with specific roles in neuron differentiation and glioblastoma suppression.* Mol Cell Biol, 2011. **31**(6): p. 1225-39.
3. Hlady, R.A., et al., *Loss of Dnmt3b function upregulates the tumor modifier Ment and accelerates mouse lymphomagenesis.* J Clin Invest, 2012. **122**(1): p. 163-77.
4. Duns, G., et al., *Histone methyltransferase gene SETD2 is a novel tumor suppressor gene in clear cell renal cell carcinoma.* Cancer Res, 2010. **70**(11): p. 4287-91.
5. Rounbehler, R.J., et al., *Tristetraprolin impairs myc-induced lymphoma and abolishes the malignant state.* Cell, 2012. **150**(3): p. 563-74.
6. Bernard, D. and D. Vindrieux, *PLA2R1: expression and function in cancer.* Biochim Biophys Acta, 2014. **1846**(1): p. 40-4.

7. Steven Siqing Wang, E.D.E., Jia Ling Li, Liying Huang, Adi Gazdar, John Minna and Glen A. Evans, *Alterations of the PPP2R1B Gene in Human Lung and Colon Cancer.* Science, 1998. **282**(5387): p. 284-287.
8. Simo-Riudalbas, L., et al., *KAT6B Is a Tumor Suppressor Histone H3 Lysine 23 Acetyltransferase Undergoing Genomic Loss in Small Cell Lung Cancer.* Cancer Res, 2015. **75**(18): p. 3936-45.
9. Martinelli, P., et al., *GATA6 regulates EMT and tumour dissemination, and is a marker of response to adjuvant chemotherapy in pancreatic cancer.* Gut, 2017. **66**(9): p. 1665-1676.
10. Lin, W., *High-Resolution Characterization Of The Leukemic Cutaneous T-Cell Lymphoma Genome.* Yale Medicine Thesis Digital Library, 2011. **1573**.
11. Jimenez, G., S.Y. Shvartsman, and Z. Paroush, *The Capicua repressor--a general sensor of RTK signaling in development and disease.* J Cell Sci, 2012. **125**(Pt 6): p. 1383-91.
12. Simon-Carrasco, L., et al., *Inactivation of Capicua in adult mice causes T-cell lymphoblastic lymphoma.* Genes Dev, 2017. **31**(14): p. 1456-1468.
13. Tan, Q., et al., *Loss of Capicua alters early T cell development and predisposes mice to T cell lymphoblastic leukemia/lymphoma.* Proc Natl Acad Sci U S A, 2018. **115**(7): p. E1511-E1519.
14. Takachi, T., et al., *Lymphoblastic lymphoma with mature b-cell immunophenotype and MLL-AF9 in a child.* Pediatr Blood Cancer, 2011. **57**(7): p. 1251-2.
15. Doherty B., C.S., Vareslija D., Charmsaz S., Bolger J., Hill A., and Young L., *ADAM22 as a therapeutic target for endocrine resistant metastatic breast cancer.* ESMO Open, 2018. **3**(Suppl 2): p. PO-507.
16. Gao, F., et al., *Global analysis of DNA methylation in hepatocellular carcinoma by a liquid hybridization capture-based bisulfite sequencing approach.* Clin Epigenetics, 2015. **7**: p. 86.
17. Heselmeyer-Haddad, K.M., et al., *Single-cell genetic analysis reveals insights into clonal development of prostate cancers and indicates loss of PTEN as a marker of poor prognosis.* Am J Pathol, 2014. **184**(10): p. 2671-86.
18. Zhang, M., et al., *Developmental pluripotency-associated 4: a novel predictor for prognosis and a potential therapeutic target for colon cancer.* J Exp Clin Cancer Res, 2015. **34**: p. 60.
19. Amini, S., et al., *The expressions of stem cell markers: Oct4, Nanog, Sox2, nucleostemin, Bmi, Zfx, Tcl1, Tbx3, Dppa4, and Esrrb in bladder, colon, and prostate cancer, and certain cancer cell lines.* Anat Cell Biol, 2014. **47**(1): p. 1-11.
20. Ambatipudi, S., et al., *Downregulation of keratin 76 expression during oral carcinogenesis of human, hamster and mouse.* PLoS One, 2013. **8**(7): p. e70688.
21. Ying Huang, D.G.B., James Y Dai, Ulrike Peters, David A Hinds, David R Cox, Erica Beilharz, Rowan T Chlebowski, Jacques E Rossouw, Anne McTiernan, Thomas Rohan and Ross L Prentice, *Genetic variants in the MRPS30 region and postmenopausal breast cancer risk.* Genome Medicine, 2011. **3**(42).
22. Kim, M.S., et al., *Neurofilament heavy polypeptide regulates the Akt-beta-catenin pathway in human esophageal squamous cell carcinoma.* PLoS One, 2010. **5**(2): p. e9003.
23. Kamata, T., *Roles of Nox1 and other Nox isoforms in cancer development.* Cancer Sci, 2009. **100**(8): p. 1382-8.

24. Wang, Y., et al., *NADPH Oxidase Activation Contributes to Heavy Ion Irradiation-Induced Cell Death.* Dose Response, 2017. **15**(1): p. 1559325817699697.
25. Girard, B.J., et al., *PELP1: a review of PELP1 interactions, signaling, and biology.* Mol Cell Endocrinol, 2014. **382**(1): p. 642-651.
26. Hart, T., et al., *High-Resolution CRISPR Screens Reveal Fitness Genes and Genotype-Specific Cancer Liabilities.* Cell, 2015. **163**(6): p. 1515-26.
27. L-W Ding, Q.-Y.S., D-C Lin, W Chien, N Hattori, X-M Dong, S Gery, M Garg, N B Doan, J W Said, J-F Xiao, H Yang, L-Z Liu, X Meng, R Y-J Huang, K Tang & H P Koeffler, *LNK (SH2B3): paradoxical effects in ovarian cancer.* Oncogene, 2015. **34**: p. 1463-1474.
28. Drolez, A., et al., *ST6GALNAC5 Expression Decreases the Interactions between Breast Cancer Cells and the Human Blood-Brain Barrier.* Int J Mol Sci, 2016. **17**(8).
29. Hirabayashi, S., et al., *ZNF384-related fusion genes define a subgroup of childhood B-cell precursor acute lymphoblastic leukemia with a characteristic immunotype.* Haematologica, 2017. **102**(1): p. 118-129.
30. Hu C., K.E., and Ray P., *Human apolipoprotein L1 (ApoL1) in cancer and chronic kidney disease.* FEBS Letters, 2012. **586**(7): p. 947-955.
31. Liu X., Z.W., Wang W., Shen H., Liu L., Lou W., Wang X., and Yang P., *A new panel of pancreatic cancer biomarkers discovered using a mass spectrometry-based pipeline.* British Journal Of Cancer. **117**.
32. Tang, B., et al., *HOXA7 plays a critical role in metastasis of liver cancer associated with activation of Snail.* Mol Cancer, 2016. **15**(1): p. 57.
33. Zhang, Y., et al., *Homeobox A7 stimulates breast cancer cell proliferation by up-regulating estrogen receptor-alpha.* Biochem Biophys Res Commun, 2013. **440**(4): p. 652-7.
34. Homig-Holzel, C., et al., *Antagonistic TSC22D1 variants control BRAF(E600)-induced senescence.* EMBO J, 2011. **30**(9): p. 1753-65.
35. Liu, T., et al., *RBFOX3 Promotes Tumor Growth and Progression via hTERT Signaling and Predicts a Poor Prognosis in Hepatocellular Carcinoma.* Theranostics, 2017. **7**(12): p. 3138-3154.
36. Qian Yang1, Michael H. Roehrl1,2,3 and Julia Y. Wang1,2,4, *Proteomic profiling of antibody-inducing immunogens in tumor tissue identifies PSMA1, LAP3, ANXA3, and maspin as colon cancer markers.* Oncotarget, 2018. **9**(3): p. 3996-4019
37. Romani, P., et al., *Extracellular NME proteins: a player or a bystander?* Lab Invest, 2018. **98**(2): p. 248-257.
38. Witkiewicz, A.K., et al., *Towards a new "stromal-based" classification system for human breast cancer prognosis and therapy.* Cell Cycle, 2009. **8**(11): p. 1654-8.
39. Kluth, G., Krohn, Weischenfeld, Tsourlakis, Paustian, Ahrary, Ahmed, Scherzai, Meyer, Sirma, Korbel, Sauter, Schlomm, Simon and Minner, *Prevalence of chromosomal rearrangements involving non-ETS genes in prostate cancer.* International Journal of Oncology, 2015. **46**: p. 1637-1642.
40. Yongjun Zhang, S.H., AiqinZhang, Xiangming Kong, Chuming Jiang, Dehou Deng, and Bao Wenlong, *Association Between Polymorphisms in COMT, PLCH1, and CYP17A1, and Non–Small-Cell Lung Cancer Risk in Chinese Nonsmokers.* Clinical Lung Cancer, 2013. **14**(1): p. 45-49.

41. Shi, T.Y., et al., *CASP7 variants modify susceptibility to cervical cancer in Chinese women.* Sci Rep, 2015. **5**: p. 9225.
42. Ng, D., et al., *Replication of a genome-wide case-control study of esophageal squamous cell carcinoma.* Int J Cancer, 2008. **123**(7): p. 1610-5.
43. Sayan, O., et al., *High Frequency of Inherited Variants in the MEFV Gene in Acute Lymphocytic Leukemia.* Indian J Hematol Blood Transfus, 2011. **27**(3): p. 164-8.
44. Kusunoki, *Overexpression of the signal peptide whirlin isoform 2 is related to disease progression in colorectal cancer patients.* International Journal of Oncology, 2009. **35**(4).
45. Li, H., et al., *The histone methyltransferase SETDB1 and the DNA methyltransferase DNMT3A interact directly and localize to promoters silenced in cancer cells.* J Biol Chem, 2006. **281**(28): p. 19489-500.
46. Zhang, Y., et al., *Rare coding variants and breast cancer risk: evaluation of susceptibility Loci identified in genome-wide association studies.* Cancer Epidemiol Biomarkers Prev, 2014. **23**(4): p. 622-8.
47. Karagiannis, G.S., et al., *Signatures of breast cancer metastasis at a glance.* Journal of Cell Science, 2016. **129**(9): p. 1751-1758.
48. Bohle, V., et al., *Role of early B-cell factor 1 (EBF1) in Hodgkin lymphoma.* Leukemia, 2013. **27**(3): p. 671-9.
49. Yachida, S., et al., *Genomic Sequencing Identifies ELF3 as a Driver of Ampullary Carcinoma.* Cancer Cell, 2016. **29**(2): p. 229-40.
50. Nair, P., et al., *Recurrent genomic imbalances in primary effusion lymphomas.* Cancer Genet Cytogenet, 2006. **171**(2): p. 119-21.
51. Scholtysik, R., et al., *Detection of genomic aberrations in molecularly defined Burkitt's lymphoma by array-based, high resolution, single nucleotide polymorphism analysis.* Haematologica, 2010. **95**(12): p. 2047-55.
52. Giona, F., et al., *Treatment-free remission after imatinib discontinuation is possible in paediatric patients with chronic myeloid leukaemia.* Br J Haematol, 2015. **168**(2): p. 305-8.
53. Lindblad O., C.R.A., Moharram S. A., Kabir N. N., Sun J., Kazi J. U., Rönnstrand L., *The role of HOXB2 and HOXB3 in acute myeloid leukemia.* Biochem Biophys Res Commun, 2015. **467**(4): p. 742-747.
54. Frohling, S., et al., *HOX gene regulation in acute myeloid leukemia: CDX marks the spot?* Cell Cycle, 2007. **6**(18): p. 2241-5.
55. Kent WJ, S.C., Furey TS, Roskin KM, Pringle TH, Zahler AM, Haussler D., *The human genome browser at UCSC.* Genome Res, 2002. **12**(6): p. 996-1006.
56. Busse, A., et al., *An intron 9 containing splice variant of PAX2.* J Transl Med, 2009. **7**: p. 36.
57. Zhang, J., et al., *The genomic landscape of mantle cell lymphoma is related to the epigenetically determined chromatin state of normal B cells.* Blood, 2014. **123**(19): p. 2988-96.
58. Richard Dillon, L.C., Alka Saxena, Rosamond Nuamah, Ghazala Mirza, Amie Jaye, Michael Simpson, Sean Whittaker, Charles R. M. Bangham, Graham P. Taylor and Paul Fields, *Whole Exome Sequencing of Flow Purified Tumour Cells Reveals Recurrently Mutated Genes and Pathways in Adult T-Cell Lymphoma/Leukaemia (ATLL).* Blood, 2015. **126**(1469).

59. Roberti, A., et al., *Type II enteropathy-associated T-cell lymphoma features a unique genomic profile with highly recurrent SETD2 alterations.* Nat Commun, 2016. **7**: p. 12602.
60. Stratton, K., et al., *Mp30-12 Renal Cell Carcinoma and Non-Hodgkin's Lymphoma: Genomic Approaches to Identification of Shared Susceptibility.* The Journal of Urology, 2014. **191**(4).
61. Park, S.K., et al., *Innate immunity and non-Hodgkin's lymphoma (NHL) related genes in a nested case-control study for gastric cancer risk.* PLoS One, 2012. **7**(9): p. e45274.
62. Elvers, I., et al., *Exome sequencing of lymphomas from three dog breeds reveals somatic mutation patterns reflecting genetic background.* Genome Research, 2015. **25**(11): p. 1634-1645.
63. Ruiz-Ballesteros, E., et al., *Splenic marginal zone lymphoma: proposal of new diagnostic and prognostic markers identified after tissue and cDNA microarray analysis.* Blood, 2005. **106**(5): p. 1831-8.
64. Zhang, J., et al., *Genetic heterogeneity of diffuse large B-cell lymphoma.* Proc Natl Acad Sci U S A, 2013. **110**(4): p. 1398-403.
65. Drazovska, M., et al., *Comparative genomic hybridization in detection of DNA changes in canine lymphomas.* Anim Sci J, 2017. **88**(1): p. 27-32.
66. McDonald J., K.A., Beheshti A., Pilichowska M., Burgess K., Ricks-Santi L., McNiel E., London C., Ravi D. and Evens A., *Comparative oncology DNA sequencing of canine T cell lymphoma via human hotspot panel.* Oncotarget, 2018. **9**(32): p. 22693-22702.
67. Sean D Hooper, X.J., Elisabeth Sundström, Farah L Rehman, Christian Tellgren-Roth, Tobias Sjöblom and Lucia Cavelier, *Sequence based analysis of U-2973, a cell line established from a double-hit B-cell lymphoma with concurrent MYC and BCL2 rearrangements.* BMC Research Notes, 2012. **5**(648).
68. Nagel, S., et al., *Transcriptional deregulation of homeobox gene ZHX2 in Hodgkin lymphoma.* Leuk Res, 2012. **36**(5): p. 646-55.
69. Pan, H., et al., *Epigenomic evolution in diffuse large B-cell lymphomas.* Nat Commun, 2015. **6**: p. 6921.
70. Zhang, Y., et al., *High KIF2A expression predicts unfavorable prognosis in diffuse large B cell lymphoma.* Ann Hematol, 2017. **96**(9): p. 1485-1491.

Supplementary Table 1: High or moderate impact, high confidence variants for case P1 (poor responder 1).

* represents previously reported association with canine lymphoma ^ represents previously reported association with human lymphoma. N+T represents a variant present in the tumour and as a heterozygous allele in the buffy coat. T represents enrichment in the corresponding gene in the tumor sample. Allele type – represents a deletion, otherwise, listed bases are insertions.

Type of indel	Location	Allele	Impact	Gene ID	Protein Name	Variant Type
N+T	14:14032479-14032482	-	moderate	ADAM22 ^[50]	ADAM metallopeptidase domain 22	Inframe Deletion
N+T	28:25816770-25816770	CGGCGGCGGCGG	moderate	ATRNL1 ^[51]	Attractin like 1	Inframe Insertion
N+T	10:26582959-26582965	-	moderate	BAIAP2L2	BAI1 associated protein 2 like 2	Inframe Deletion
N+T	14:56585850-56585856	-	moderate	CTTNBP2 ^[52]	Cortactin binding protein 2	Inframe Deletion
N+T	17:19863775-19863776	-	high	DTNB	Dystrobrevin beta	Frameshift
N+T	37:1433815-1433815	GGGCGC	moderate	GLS	Glutaminase	Inframe Insertion
N+T	X:76315044-76315048	-	high	GPRASP1 ^[51]	G protein-coupled receptor associated sorting protein 1	Frameshift
N+T	4:65159988-65159988	CCGCAGCCC	moderate	HCN1	Hyperpolarization activated cyclic nucleotide gated potassium channel 1	Inframe Insertion, Splice Region
T only	9:24812827-24812827	CCT	moderate	HOXB3 ^[53, 54]	Homeobox B3	Inframe Insertion
N+T	27:2345999-2345999	GCAGCAGCGGAGGCTACCAGAGTG	moderate	KRT76	Uncharacterized protein	Inframe Insertion
N+T	28:36918697-36918697	CTT	moderate	MKI67	Marker of proliferation Ki-67	Inframe Insertion
N+T	4:65536829-65536829	GCGGGC	moderate	MRPS30	Mitochondrial ribosomal protein S30	Inframe Insertion
N+T	26:22732974-22732998	-	high	NEFH*[55]	Neurofilament heavy	Splice Donor, Coding Sequence, Intron Variant
N+T	26:22732974-22732998	-	moderate	NEFH*[55]	Neurofilament heavy	Inframe Deletion
N+T	28:33808713-33808713	CGCCGCCGC	moderate	NKX1-2	NK1 homeobox 2	Inframe Insertion

N+T	1:45169147-45169147	AAAAC	high	NOX3	NADPH oxidase 3	Frameshift
N+T	28:13514398-13514399	-	high	PAX2^[56]	Paired box 2	Splice Acceptor Variant
N+T	20:18857742-18857742	GCGGC A	moderate	PDZRN3	PDZ domain containing ring finger 3	Inframe Insertion
T only	5:31859396-31859402	-	moderate	PELP1	Proline, glutamate and leucine rich protein 1	Inframe Deletion
N+T	14:18121396-18121399	-	moderate	RBM48	RNA binding motif protein 48	Inframe Deletion
N+T	33:4985282-4985282	CGCTG GCGGG GA	moderate	RIOX2	Ribosomal oxygenase 2	Inframe Insertion
T only	1:114475221-114475222	-	high	RYR1 ^[57]	Ryanodine receptor 1	Frameshift
N+T	20:55172220-55172220	CCGCC CCCGC CG	moderate	SEMA6B ^[10]	Semaphorin 6B	Inframe Insertion
N+T	26:9069232-9069236	-	high	SH2B3 ^[58, 59]	SH2B adaptor protein 3	Frameshift
T only	2:73738989-73738990	-	moderate	SH3BGRL3 ^[10]	SH3 domain binding glutamate rich protein like 3	Frameshift
N+T	6:69866893-69866896	-	moderate	ST6GALNAC5	ST6 N-acetylgalactosaminide alpha-2,6-sialyltransferase 5	Inframe Deletion
N+T	10:17023385-17023385	C	high	TUBGCP6	Tubulin gamma complex associated protein 6	Frameshift
N+T	24:32910785-32910786	-	high	WFDC10A	WAP four-disulfide core domain 10A	Frameshift
N+T	27:38367848-38367878	-	moderate	ZNF384 ^[60]	Zinc finger protein 384	Inframe Deletion

Supplementary Table 2: High or moderate impact, high confidence variants for case P2 (poor responder 2).

* represents previously reported association with canine lymphoma ^ represents previously reported association with human lymphoma. N+T represents a variant present in the tumour and as a heterozygous allele in the buffy coat. T represents enrichment in the corresponding gene in the tumor sample. Allele type – represents a deletion, otherwise, listed bases are insertions.

Type of indel	Location	Allele	Impact	Gene ID	Protein Name	Variant Type
T only	1:112085161-112085162	-	high	CIC^[12, 13](mice)	Capicua transcriptional repressor	Frameshift
N+T	1:72928059-72928062	-	moderate	GAS1	Growth arrest specific 1	Inframe Deletion
N+T	1:118924872-118924874	-	high	LRP3	LDL receptor related protein 3	Frameshift, Splice Region
T only	1:93037348-93037348	GCGGCGGCGGCGGCGGCGGCG	moderate	PLPP6	Phospholipid phosphatase 6	Inframe Insertion

Supplementary Table 3: High or moderate impact, high confidence variants for case P3 (poor responder 3).

* represents previously reported association with canine lymphoma ^ represents previously reported association with human lymphoma. ~ represents previously reported to not be associated with human lymphoma. N+T represents a variant present in the tumour and as a heterozygous allele in the buffy coat. T represents enrichment in the corresponding gene in the tumor sample. Allele type – represents a deletion, otherwise, listed bases are insertions.

Type of indel	Location	Allele	Impact	Gene ID	Protein Name	Variant Type
N+T	28:25816770-25816770	CGGCG GCGGC GG	Moderate	ATRNL1	Attractin like 1	Inframe Insertion
N+T	10:26582959-26582965	-	Moderate	BAIAP2L2	BAI1 associated protein 2 like 2	Inframe Deletion
N+T	28:24623398-24623398	GTGTG T	Moderate	CASP7^[61]	Caspase 7	Protein Altering, Splice Region
T only	4:9877424-9877439	-	Moderate	CCSAP	Zinc finger protein 384	Inframe Deletion
N+T	5:32592363-32592363	C	High	EFNB3	Ephrin b3	Frameshift
N+T	1:81543455-81543455	G	High	FOXB2	Forkhead box B2	Frameshift
N+T	25:50490983-50490983	C	High	GPC1	Glypican 1	Frameshift
N+T	9:19078030-19078040	-	High	GRN	Granulin precursor	Frameshift
N+T	25:49318269-49318270	-	High	HDAC4	Histone deacetylase: deacetylation of lysine residues on N-terminal part of the core histones	Frameshift
T only	14:40329189-40329192	-	Moderate	HOXA7 *[55, 62]	Homeobox A7	Inframe Deletion
N+T	2:77366534-77366535	-	High	HSPG2	Heparan sulfate proteoglycan 2	Frameshift
T only	4:25292801-25292825	-	High	KAT6B	Histone acetyltransferase	Splice Acceptor, Coding Sequence, Intron
N+T	9:21867315-21867339	-	Moderate	KRT10	Ketain, type 1 cytoskeletal 10: epidermal barrier on plantar skin	Inframe Deletion
N+T	24:15803269-15803269	CGGC CT	Moderate	LRRN4	Leucine rich repeat neuronal 4	Inframe Insertion
T only	5:77412270-77412271	-	High	MARVELD3	MARVEL domain containing 3	Frameshift
N+T	6:37982406-37982407	-	High	MEFV ^[43]	Pyrin innate immunity regulator	Frameshift

T only	11:40000277-40000280	-	Moderate	MLLT3 (AF9) *[55, 62] ^[14]	MLLT3, super elongation complex subunit (Protein AF-9)	Inframe Deletion
N+T	33:29046187-29046187	T	High	MUC20	Mucin 20, cell surface associated	Frameshift
N+T	30:755845-755857	-	Moderate	NUTM1	NUT midline carcinoma family member 1	Inframe Deletion
N+T	20:46726307-46726311	-	High	PGLYRP2	Peptidoglycan recognition protein 2	Frameshift, Splice Region
T only	36:5793614-5793614	A	High	PLAZR1	Phospholipase A2 receptor 1	Frameshift
T only	23:49281776-49281776	CGGGG GCGGG GCCTC CGGGG GCGGG GTCTC	Moderate	PLCH1	Phosphoinositide phospholipase C	Inframe Insertion
N+T	20:5585939-5585939	T	High	PLXND1	Plexin D1	Frameshift, Splice Region
N+T	5:21323892-21323898	-	Moderate	PPP2R1B	Protein phosphatase 2 scaffold subunit Abeta	Inframe Deletion
N+T	20:48517884-48517886	-	High	RFX1	Regulatory factor x1	Frameshift
N+T	9:51924902-51924905	-	Moderate	SETX * [55, 62] ^[63]	Senataxin	Inframe Deletion
N+T	6:69866825-69866831	-	Moderate	ST6GALNAC5	ST6 N-acetylgalactosaminide alpha-2,6-sialyltransferase 5	Inframe Deletion
N+T	2:72910715-72910716	-	High	SYTL1	Synaptotagmin like 1	
N+T	6:31471866-31471867	-	High	TNP2	Nuclear transition protein 2	Frameshift
N+T	17:40080828-40080828	G	High	TRABD2A	TraB domain containing 2A	Frameshift
N+T	23:8811022-8811022	G	High	TTC21A	Tetratricopeptide repeat domain 21a	Frameshift
N+T	24:20540696-20540699	-	Moderate	ZCCHC3	zinc-finger CCHC-type containing 3	Inframe Deletion
N+T	1:113816754-113816758	-	High	ZFP36^[5]	ZFP36 ring finger protein	Frameshift
T only	27:38367811-38367811	GGCCC AGGCC CAGGC CCA	Moderate	ZNF382	Centriole, cilia and spindle associated protein	Inframe Deletion
T only	4:25586589-25586590	-	High	ZNF503	Zinc finger protein 503	Frameshift

Supplementary Table 4: High or moderate impact, high confidence variants for case G1 (good responder 1).

* represents previously reported association with canine lymphoma ^ represents previously reported association with human lymphoma. N+T represents a variant present in the tumour and as a heterozygous allele in the buffy coat. T represents enrichment in the corresponding gene in the tumor sample. Allele type – represents a deletion, otherwise, listed bases are insertions.

Type of indel	Location	Allele	Impact	Gene ID	Protein Name	Variant Type
N+T	20:50958383-50958383	GGCGGGCCCGGG	moderate	ANGPTL6	Angiopoietin like 6	Inframe Insertion
N+T	5:41480390-41480390	CAG	moderate	ATPAF2	ATP synthase mitochondrial F1 complex assembly factor 2	Inframe Insertion
N+T	1:110148630-110148633	-	moderate	CD3EAP	CD3e molecule associated protein	Inframe Deletion
N+T	30:7641439-7641439	CGCCGCCCGCCGCCCGCCGCCCGCC	high	CHST14	Carbohydrate sulfotransferase	Frameshift
N+T	13:61888285-61888307	-	high	COX18	Cytochrome c oxidase assembly factor	Frameshift
N+T	21:29937061-29937069	-	high	DCHS1	Dachsous cadherin-related 1	Frameshift
N+T	2:48723570-48723572	-	high	DIMT1	rRNA adenine N(6)-methyltransferase	Frameshift
N+T	24:22096444-22096444	G	high	DNMT3B^ [3]	DNA methyltransferase 3 beta	Frameshift
N+T	8:31109087-31109087	CCGCCGCCGCCG	moderate	FBXO34	F-box protein 34	Frameshift
T only	14:40329189-40329192	-	moderate	HOXA7*[55, 62]	Homeobox A7	Inframe Deletion
N+T	7:29257443-29257445, 7:29272928-29272932	-	high	NME7	NME/NM23 family member 7	Frameshift
N+T	7:29258375-29258378	-	moderate	NME7	NME/NM23 family member 7	Inframe Deletion
N+T	16:33560543-33560545	-	high	PPP2CB	Serine/threonine-protein phosphatase	Frameshift
N+T	20:8332662-8332662	CCCCGC	moderate	PRRT3	Proline rich transmembrane protein 3	Inframe Insertion
T only	9:2369722-2369722	C	high	RBFOX3	RNA binding protein fox-1 homolog	Frameshift

T only	20:41712352-41712352	G	high	SETD2 *[62] ^[59, 64]	SET domain containing 2	Frameshift
N+T	16:47445467-47445467, 16:47445530-47445531	TTTGG GAACA GGGG	high	TENM3	Teneurin transmembrane protein 3	Frameshift
N+T	16:47445471-47445471	GGG	moderate	TENM3	Teneurin transmembrane protein 3	Inframe Insertion
N+T	17:31696378-31696378	G	high	TMEM178A	Transmembrane protein 178A	Frameshift, Splice Region
T only	5:32563439-32563439	TAGTC AACTA TAG	high	TP53 *[62, 65] ^[59, 66]	Cellular tumor antigen p53	Frameshift
N+T	22:6375934-6375940	-	moderate	TSC22D1	TSC22 domain family member 1	Inframe Deletion
T only	20:55237167-55237167	T	high	UBXN6	UBX domain protein 6	Frameshift, Splice Region

Supplementary Table 5: High or moderate impact, high confidence variants for case G3 (good responder 3).

* represents previously reported association with canine lymphoma ^ represents previously reported association with human lymphoma. N+T represents a variant present in the tumour and as a heterozygous allele in the buffy coat. T represents enrichment in the corresponding gene in the tumor sample. Allele type – represents a deletion, otherwise, listed bases are insertions.

Type of indel	Location	Allele	Impact	Gene ID	Protein Name	Variant Type
T only	20:37566285-37566291	-	Moderate	ALAS1	5'-aminolevulinate synthase 1	Inframe Deletion
N+T	21:47390077-47390077	A	High	ANO3 ^[67]	Anoctamin	Stop Gained, Frameshift, Splice Region
T only	28:10853503-10853566	-	High	C10orf62	Chromosome 10 open reading frame 62	Splice Acceptor, Coding
T only	17:22910433-22910434	-	High	C17H2orf71	Uncharacterized protein	Frameshift
N+T	13:61887908-61887917	-	Moderate	COX18	Cytochrome c oxidase assembly factor	Inframe Insertion
T only	29:16711889-16711934	-	High	CSPP1	Centrosome and spindle pole associated protein 1	Frameshift
T only	X:43109757-43109758	-	Moderate	DGKK	Diacylglycerol kinase	Inframe Deletion
N+T	17:19530692-19530693	-	High	DNMT3A ^[66]	DNA methyltransferase 3 alpha	Frameshift
T only	4:51379332-51379334	-	High	EBF1 ^[48]	Early B-cell factor 1	Frameshift
N+T	20:12472686-12472708	-	High	EDEM1 ^[68]	alpha-1,2-Mannosidase	Splice Donor, Coding Sequence, Intron
N+T	21:44256224-44256230	-	Moderate	FANCF	Fanconi anemia complementation group F	Inframe Deletion
N+T	27:5097837-5097837	ACTGTAG	High	KCNH3 ^[69]	Potassium voltage-gated channel subfamily H member 3	Frameshift
N+T	7:14426348-14426348	AGGCG GTGC	Moderate	KIAA1614	Uncharacterized protein	Inframe Insertion
T only	2:48645386-48645386	C	High	KIF2A ^[70]	Kinesin-like protein	Frameshift
T only	7:44757831-44757837	-	Moderate	LOXHD1 *[55, 62]	Lipoxygenase homology domains 1	Inframe Deletion
T only	2:74303945-74303945	T	High	MAN1C1 ^[10]	alpha-1,2-Mannosidase	Frameshift, Splice
N+T	5:57227123-57227123	G	High	MORN1	MORN repeat containing 1	Frameshift
T only	26:22732974-	-	High	NEFH	Neurofilament heavy	Splice Acceptor,

	22732998					Coding Sequence, Intron
T only	26:22732974-22732998	-	Moderate	NEFH	Neurofilament heavy	Inframe Deletion
N+T	1:71290507-71290507	A	High	PTCH1	Patched 1	Splice Donor, Frameshift
N+T	38:1747783-1747792	-	Moderate	TMCC2	Transmembrane and coiled-coil domain family 2	Inframe Deletion
N+T	12:71937263-71937270	-	High	TSPYL1	TSPY like 1	Frameshift
T only	17:53216419-53216421	-	High	VANGL1	Vang-like protein	Splice Donor, Coding Sequence, Intron
N+T	11:68699669-68699675	-	Moderate	WHRN	Whirlin	Inframe Insertion
